# Long Term Results of the Modified Bentall Procedure With Mechanical and Biological Composite Valve Grafts

**DOI:** 10.3389/fcvm.2022.867732

**Published:** 2022-04-06

**Authors:** Paul Werner, Jasmin Gritsch, Alexandra Kaider, Iuliana Coti, Emilio Osorio, Stephane Mahr, Marie-Elisabeth Stelzmueller, Alfred Kocher, Günther Laufer, Martin Andreas, Marek Ehrlich

**Affiliations:** ^1^Department of Cardiac Surgery, Medical University of Vienna, Vienna, Austria; ^2^Center for Medical Statistics, Informatics and Intelligent Systems, Medical University of Vienna, Vienna, Austria

**Keywords:** modified Bentall procedure, composite valve graft replacement, valve-related adverse events, aortic valve replacement, aortic replacement

## Abstract

**Objectives:**

Despite the evident shift toward biological prostheses, the optimal choice of valve remains controversial in composite valve graft (CVG) replacement. We investigated long-term morbidity and mortality after CVG implantation in an all-comer cohort with a subgroup analysis of patients aged 50–70 years stratified after valve type.

**Methods:**

A total of 507 patients underwent the Bentall procedure with either a mechanical (MCVG, *n* = 299) or a biological (BCVG *n* = 208) CVG replacement between 2000 and 2020. A single-center analysis comprising clinical and telephone follow-up was conducted to investigate late mortality and morbidity

**Results:**

The 30-day mortality in all patients [age 56 ± 14 years, 78.1% male, EuroSCORE II 3.12 (1.7; 7.1)] was 5.9%. Patients who were electively operated on had a 30-day mortality of 1.5% (*n* = 5) while it remained higher in urgent/emergent procedures (*n* = 25, 15.4%). Survival at 10 and 15 years was 78.19 ± 2.26% and 72.6 ± 3.2%, respectively. In patients aged 50–70 years (*n* = 261; MCVG = 151, BCVG = 110), survival did not differ significantly between the valve groups (*p* = 0.419). Multivariable analysis showed no significant impact of valve type on survival (*p* = 0.069). A time-varying relation with survival was notable, showing a higher risk in the MCVG group in the early postoperative phase, which declined compared to the BCVG group in the course of follow-up.

**Conclusions:**

The Bentall technique presents with excellent mortality when performed electively. The type of valve prosthesis showed no statistically significant effect on mortality in patients aged 50–70 years. However, a time-varying relation showing an initially higher risk with MCVG which decreased compared to BCVG at long-term follow-up was notable. Further studies with even longer follow-up of BCVGs will clarify the ideal choice of prosthesis in this patient subset.

## Introduction

Initially, described in 1968 by Bentall and De Bono, composite valve graft (CVG) replacement, also known as the Bentall procedure, represented a novel surgical option for dilated aortic roots (1). The procedure underwent important modifications including, most notably, the abandonment of the wrap-inclusion technique due to albumin-coated Dacron prostheses and the implementation of the coronary-button technique (2) and has become a widely adopted standard of care for various root pathologies (3–5).

Although valve-sparing solutions are often the preferred approach in selected cases, CVG implantation remains universally applicable in an all-comer cohort and is not limited to favorable valve morphology. While earlier reports of patients with CVG only included mechanical conduits (5), the evident trend in surgical aortic valve replacement (SAVR) toward increased use of bioprosthetic valves has also led to a rising number of biological CVG implantations. However, the problem of the optimal choice of prosthesis in SAVR remains controversial in CVG replacement. Only a few studies have investigated the effect of the type of CVG on postoperative mortality and morbidity, and they have shown no relevant differences in postoperative mortality (6–8).

Herein, we report data on 507 patients with long-term follow-up and 21 years of experience with the Bentall procedure in a tertiary care center. Given the controversy regarding the optimal choice of conduit, a subgroup analysis of patients aged 50–70 years was performed to investigate the impact of valve choice on survival after CVG replacement.

## Materials and Methods

### Ethics Statement

The following study was reviewed and approved by the ethical committee of the Medical University of Vienna (ethical-board number 2311/2020; date of approval 19.01.2021).

### Patients

All patients who underwent aortic valve and associated aortic surgery between January 2000 and December 2021 were screened for their surgical procedure to identify patients who underwent CVG implantation after the modified Bentall technique. Patients who underwent root replacement that did not use CVGs and patients younger than 18 years were excluded. Retrospective data were obtained *via* the institutional database and prospective telephone follow-up was performed with assessment of adverse events (AEs) and reinterventions. Our institutional database is part of the Austrian quality-control system for cardiac surgery and is being monitored on a yearly basis. The stored mortality data are in concordance with the Austrian Federal Statistical-Agency and are updated annually.

### Endpoint Definitions

The primary endpoint of this study was mortality in the overall cohort and subgroup of patients aged 50–70 years. Postoperative AEs were defined after the current Society of Thoracic Surgeons (STS)/American Association of Thoracic Surgery (AATS)/European Association for Cardio-Thoracic Surgery (EACTS) guidelines for reporting mortality and morbidity after cardiac valve interventions and were grouped into structural valve deterioration (SVD), non-structural valve dysfunction (NSVD), valve thrombosis, embolism, and bleeding events (9). Permanent neurological deficits which occurred when the patient emerged from anesthesia after the index operation were classified as perioperative stroke. Re-exploration for bleeding was classified as bleeding revision.

### Surgical Technique and Conduit Selection

All cases in this study underwent the button Bentall technique, a modification of the original Bentall procedure (1), described by Kouchoukos et al. (2).

Surgical access over a median sternotomy was established. If necessary, right subclavian artery or femoral cannulation was performed before sternotomy. After the establishment of cardiopulmonary bypass and conduction of cardioplegic arrest with cold-blood cardioplegia, aortic root dissection was performed, the coronary buttons were mobilized, and the sinuses of Valsalva were excised. When a non-prefabricated graft was used, a CVG was composed by suturing the valve into a Dacron prosthesis (3–5 mm larger than valve size) with 5–0 polypropylene sutures in a running fashion. Following the first institutional use of a Valsalva conduit in 2006, the GelweaveTM Valsalva prosthesis (Terumo Aortic, Glasgow, UK) became the preferred option for non-prefabricated CVGs. Braided 2–0 polyester sutures with pledgets were placed from the ventricular to the aortic portion of the annulus to anchor the CVG. The second line of 2–0 sutures with pledgets anchoring the graft from the outside was performed for hemostatic reasons. The left coronary button was anastomosed to the CVG in an end-to-side fashion with a 6–0 polypropylene suture. The graft was subsequently pressurized with antegrade cardioplegia to check for bleeding and to distend the graft for correct evaluation of the right coronary artery (RCA) insertion before completion of the anastomosis. A thin strip of the autologous pericardium was routinely included in the button anastomoses for better hemostasis. Distal aortic anastomosis and concomitant procedures were performed according to the underlying pathology.

The valve choice was based on the patient's age, need for anticoagulation, and preference. Patients below 60 years of age more frequently received a mechanical CVG while patients over the age of 65 years routinely received biological CVGs. Some patients under the age of 60 opted for biological CVGs to avoid lifelong anticoagulation although they were advised about the risk of valve deterioration.

### Statistical Methods

Continuous variables were described by mean (± standard deviation) or median (quartiles) in case of non-normal distributions and compared between groups of patients using the two-sample *t*-test or the Wilcoxon rank-sum test, respectively. Categorical data were compared using the chi-squared test or Fisher's exact test (if expected cell frequencies were <5). The median follow-up times were calculated using the inverse Kaplan-Meier method (10).

The Kaplan-Meier method was used to calculate the cumulative survival probability (with 95% confidence intervals) of the study population. To compare the survival of the study cohort with an age- and sex-matched standard population, mortality data from 2013 (Austrian Federal Statistical-Agency “Statistics Austria”) were used (11). The hypothetical cumulative survival of this age-sex-matched standard population was calculated using the life-table method. The comparison of survival of the study cohort to the standard population at time points of 2, 5, and 10 years was performed by z tests (standard population's survival as null hypothesis value).

To evaluate the potential valve type effect on survival, univariable and multivariable Cox proportional-hazards regression models were performed. The prognostic factors of age, EuroSCORE II (log_2_-transformed), reoperative status, and high-risk indication were included in the multivariable model in addition to the factor valve type (mechanical/biological) to account for imbalances with respect to these well-known confounding factors. Time-varying effects were tested in the Cox regression models to check the proportional hazards assumption. Since a statistically significant time-varying valve type effect was detected, this term was additionally included in the univariable and multivariable models. Directly adjusted survival curves are depicted to illustrate the multivariable adjusted valve type effect on survival. Survival times were censored after 10 years of follow-up to achieve comparable follow-up periods in these two cohorts.

Cumulative incidence functions were estimated to quantify the probability of valve-related adverse events (composite endpoint incorporating bleeding, thrombosis, embolization, and SVD) accounting for death as a competing event. Gray's test was used for group comparisons.

Two-sided *p* < 0.05 were considered statistically significant. The software SAS (SAS Institute Inc., 2016, Cary, NC, USA) was used for statistical calculations.

## Results

### Characteristics and Procedural Details

**Figure 1:**
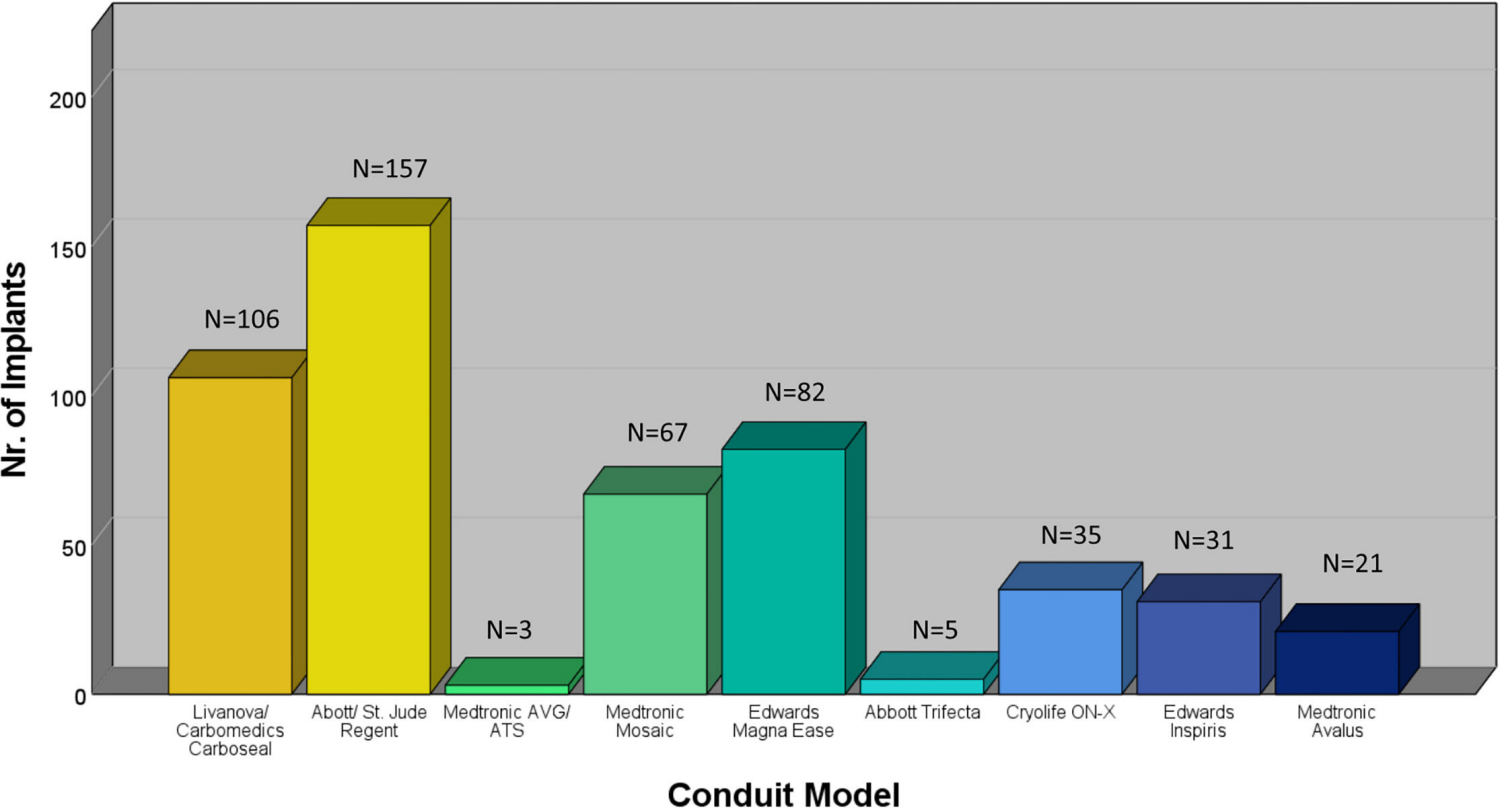
Prosthetic valve models used for composite graft implantation in the study cohort.

Between January 2000 and December 2020, 507 patients underwent a modified Bentall procedure at our institution. The mean age in the overall cohort was 56 ± 14 years and 78.1% were male. The median (quartiles) EuroSCORE II was 3.12 (1.7; 7.1) and the median STS risk of mortality was 0.89 (0.63; 1.47). Patient characteristics are summarized in Table 1. Elective surgery was performed in 68% (*n* = 345) of patients. The indications for surgery were anulo-aortic ectasia (*n* = 374; 73.8%), aortic dissection (*n* = 104; 20.5%), endocarditis (*n* = 16; 3.2%), and others (*n* = 13; 2.6%). Biological CVGs and mechanical CVGs (MCVGs) were implanted in 208 (41%) and 299 patients (59%), respectively. Prosthetic conduits are listed in Figure 1. Implantation of MCVG was performed in 86.3% (*n* = 132) of cases between 2000 and 2010 and in 47.2% (*n* = 167) of cases between 2011 and 2020 (*p* < 0.001). Procedural details are summarized in Table 2.

**Table 1:** Patient characteristics.

**Variables**	**Overall cohort**	**50–70a**		
	***n* = 507**	**MCVG (*n* = 151)**	**BCVG (*n* = 110)**	***p*-value (50–70a)**
Age (years)	56 ± 14	58 ± 6	64 ± 1	<0.001^a^
Sex (male)	396 (78.1%)	126 (83.4%)	89 (80.9%)	0.60^b^
BMI (kg/m^2^)	27 ± 4.4	27.7 ± 4.3	28.2 ± 4.5	0.41^a^
EuroSCORE II (%)	3.12 [1.7; 7.1]	2.2 [1.6–5.2]	3.45 [2.2; 7.03]	<0.001^c^
STS Prom (%)	0.89 [0.63; 1.47]	0.84 [0.61; 1.37]	0.87 [0.69; 1.40]	0.09^c^
Arterial hypertension	408 (80.5%)	127 (84.1%)	98 (89.1%)	0.25^b^
Diabetes	33 (6.5%)	10 (6.6%)	12 (10.9%)	0.22^b^
Dyslipidemia	264 (52.1%)	77 (51%)	67 (60.9%)	0.11^b^
Peripheral vascular disease	13 (2.6%)	4 (2.6%)	0	0.14^d^
Chronical lung disease	33 (6.5%)	13 (9.2%)	5 (5%)	0.23^b^
Atrial fibrillation/flutter	82 (16.2%)	29 (19.2%)	15 (13.6%)	0.24^b^
History of smoking	177 (34.9%)	48 (31.8%)	44 (40.0%)	0.17^b^
Preoperative creatinine (mg/dl)	1.06 ± 0.53	1.0 [0.85; 1.18]	0.97 [0.86; 1.15]	0.54^c^
Connective tissue disease	25 (4.9%)	4 (2.6%)	1 (0.9%)	0.31^d^
Previous cardiac surgery	64 (12.6%)	16 (10.6%)	13 (11.8%)	0.76^b^
Bicuspid valve	194 (38.3%)	64 (43.8%)	29 (26.4%)	0.004^b^

a *Two-sample t-test,*

b *Chi-squared test,*

c *Wilcoxon-rank-sum test,*

d *Fisher's-exact test*.

**Table 2:** Procedural details.

**Variables**	**Overall cohort**	**50–70a**		
		**MCVG (*n* = 151)**	**BCVG (*n* = 110)**	***p*-value (50–70a)**
**Operative status**				**0.24^a^**
Elective	345 (68.1%)	97 (64.2%)	80 (72.7%)	
Urgent	66 (13%)	18 (11.9%)	11 (10%)	
Emergent	95 (18.7%)	36 (23.8%)	18 (16.4%)	
Salvage	1 (0.2%)	0	1 (0.9%)	
**Indication**				**0.34^a^**
Anulo-aortic ectasia	374 (73.8%)	106 (70.2%)	85 (77.3%)	
Dissection	104 (20.5%)	36 (23.8%)	20 (18.2%)	
Endocarditis	17 (3.4%)	7 (4.6%)	2 (1.8%)	
Other	12 (2.4%)	2 (1.3%)	3 (2.7%)	
Concomitant procedures	189 (37.3%)	54 (35.8%)	42 (38.2%)	0.69^b^
Coronary bypass	88 (17.4%)	21 (13.9%)	12 (19.1%)	0.26^b^
Mitral	33 (6.5%)	9 (6%)	6 (5.5%)	0.87
Tricuspid	9 (1.8%)	2 (1.3%)	3 (2.7%)	0.41
Atrial-Fibrillation	10 (2%)	3 (2%)	4 (3.6%)	0.42
**Aortic**			
Hemiarch	73 (14.4%)	26 (17.2%)	16 (14.5%)	0.56^b^
Total-Arch	22 (4.3%)	5 (3.3%)	4 (3.6%)	1.0^a^
Elephant-Trunk	6 (1.2%)	0	2 (1.8%)	0.18^a^
Circulatory arrest	198 (39.1%)	60 (39.7%)	46 (41.8%)	0.74^b^
Aortic cross-clamp (min)	131 [107; 164]	126.5 [103; 158]	135 [115; 174]	0.02^c^
Extracorporeal circulation (min)	184 [150; 235]	174 [139; 226]	192.5 [161; 236]	0.02^c^

a *Fisher's-exact test*.

b *Chi-squared test*.

c *Wilcoxon-rank-sum test*.

The median follow-up for the primary endpoint survival was 81.5 (35.5; 127.1) months with 3,129 patient-years and a maximum of 243 months. The median follow-up for AEs was 47.9 (5.1; 104.7) months with a total of 2,230 patient years. In patients aged 50–70 years, the median survival follow-up was 102.8 months in the MCVG group (57.8 months for AEs) and 55.1 months in the biological CVG (BCVG) group (37.2 months for AEs).

### Post-operative Mortality

**Figure 2:**
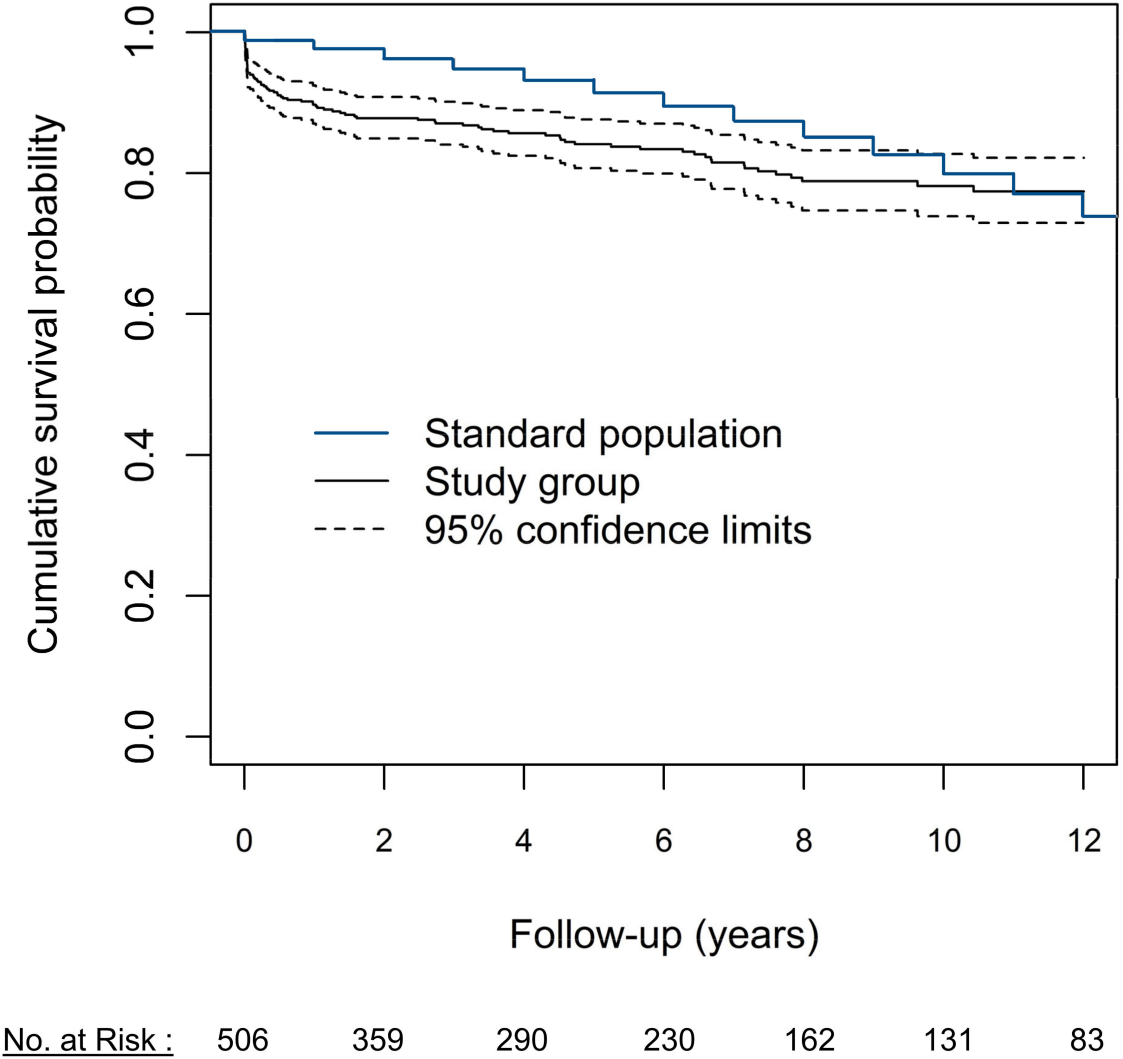
Survival of the overall cohort compared to an age- and sex-matched Austrian standard population.

The 30-day mortality in all patients was 5.9% (*n* = 30) with one case of intraprocedural mortality (0.2%). No significant difference in 30-day mortality was observed between patients who received a MCVG (*n* = 18) vs. BCVG (*n* = 12, *p* = 0.9). Patients who underwent surgery for acute type-A dissection presented with a higher 30-day mortality (*n* = 21, 20.2%, *p* < 0.001), as did patients who underwent urgent/emergent procedures (*n* = 25, 15.4%, *p* < 0.001). Patients who underwent elective surgery had a low 30-day mortality rate of 1.5% (*n* = 5). Survival at 5, 10, and 15 years was 84.1 ± 1.8, 78.2 ± 2.3, and 72.6 ± 3.2%, respectively. Our study cohort's survival was compared to an age- and sex-matched Austrian standard population (Figure 2). Survival was significantly lower in patients who underwent CVG implantation than in a standard population at 2 years (97.6 vs. 87.8; *p* < 0.0001) and 5 years (93.2 vs. 84.1; *p* < 0.0001) but not at 10 years (82.6 vs. 78.2; *p* = 0.12).

### Morbidity and Repeat Surgery

Overall, 37 patients (7.3%) received temporary mechanical circulatory support within 30 days after surgery, with 34 cases of extracorporeal membrane-oxygenation (ECMO) implantation (*n* = 6.7%) and two cases (0.4%) of intra-aortic balloon pump implantation. Indications for ECMO implantation in the patients receiving a BCVG were severely reduced left ventricular function (*n* = 2) after CBP, left ventricular failure after ventricular rupture (*n* = 1), biventricular failure (*n* = 4) due to myocardial stunning, right ventricular failure (*n* = 2), combined respiratory and hemodynamic instability (*n* = 2), and cardiopulmonary resuscitation with pulseless electrical activity at the 29th postoperative day (POD). Of those patients, two patients died within 30 days after the index surgery.

Revision surgery for bleeding or tamponade was necessary in 45 patients (re-exploration *n* = 33, 6.5%; subxiphoidal drainage *n* = 12, 2.4%). Perioperative stroke was observed in 24 cases (4.7%) and perioperative transient-ischemic attacks (TIAs) occurred in 4 patients (0.8%). Early permanent pacemaker implantation (<14 days postoperative) was necessary for 13 patients (2.3%). Two patients (1% BCVG) presented with SVD after receiving a BCVG, and NSVD was observed in one case (0.2%) with severe paravalvular dehiscence after implantation of an MCVG (the patient eventually died due to prohibitive surgical risk). Valve thrombosis was diagnosed in two patients (0.4%) with an MCVG, and surgical extirpation was required in both cases. Embolic events were observed in 27 patients (5.3%), of which 24 (4.7%; stroke *n* = 19, TIA *n* = 5) presented as cerebral and three (0.6%) as peripheral. Bleeding events were reported in 32 patients (6.3%), stratified into 12 cerebral and 20 peripheral bleeding events. Endocarditis was observed in 19 patients (3.7%), of whom 9 (1.8%) underwent root rereplacement.

Repeat surgery on the thoracic aorta, aortic valve, or coronaries was performed in 35 patients (6.9%). Valve-related reoperations (*n* = 9; 1.8%) were performed due to endocarditis requiring root rereplacement in 8 cases (1.6%) and valve thrombus requiring extirpation in one case (0.2%). Eighteen patients (3.7%) underwent aortic-related repeat surgery with 2 cases (0.4%) of root rereplacement, 1 case (0.2%) of sinotubular-junction pseudoaneurysm, 5 cases (1%) of arch replacement, 9 cases (1.8%) of thoracic endovascular aortic repair, and one case (0.2%) of thoracoabdominal replacement. Coronary-related reoperations (*n* = 8; 1.6%) were performed due to coronary button pseudoaneurysm (*n* = 3; 0.6%), kinking of the proximal RCA (*n* = 2; 0.4%), kinking of a vein-graft to the RCA (*n* = 1; 0.2%), necessity of bypass revision with simultaneous thrombus extirpation and ventricular assist-device implantation (*n* = 1; 0.2%), and ischemic cardiogenic failure (*n* = 1; 0.2%). Second repeat surgery was performed in seven patients (1.4%) with three cases (0.6%) of root rereplacement and four cases of aortic procedures (0.8%). Altogether, rereplacement of the root was performed in 13 patients (2.6%) in the overall cohort. The annualized event rate of root rereplacement was 0.6% in all patients. Postoperative mortality and morbidity are summarized in Table 3.

**Table 3:** Post-operative morbidity and mortality.

**Clinical events**	**Overall cohort**	**50–70a**		
		**MCVG (*n* = 151)**	**BCVG (*n* = 110)**	***p*-value (50–70a)**
30-day mortality	30 (5.9%)	11 (7.3%)	5 (4.5%)	0.36
ECMO (<30 days)	34 (6.7%)	4 (2.6%)	12 (10.9)	0.003
Perioperative stroke	24 (4.7%)	8 (5.3%)	8 (7.3%)	0.51^c^
Early pacemaker (<14 days)	13 (2.6%)	4 (2.6%)	3 (2.7%)	1.0^d^
Bleeding revision/drainage	45 (8.9%)	5 (3.3%)	13 (11.8%)	0.007^c^
Root rereplacement^a^	0.6% ppy (*n* = 13)	0.6% ppy (*n* = 4)	0.3% ppy (*n* = 1)	
SVD^a^	<0.01% ppy (*n* = 2)	0% ppy	0.3% ppy (*n* = 1)	
NSVD^a^	<0.01% ppy (*n* = 1)	0% ppy	0% ppy	
Embolic events^a^	1.2% ppy (*n* = 27)	2% ppy (*n* = 13)	1.1% ppy (*n* = 4)	
Bleeding^a^	1.4% ppy (*n* = 32)	1.4% ppy (*n* = 9)	1.7% ppy (*n* = 6)	
Endocarditis^a^	0.9% ppy (*n* = 19)	0.8% ppy (*n* = 5)	1.1% ppy (*n* = 4)	
Bleeding/embolization/thrombus/SVD^b^	/			0.70^e^
1 year		7.9% [4–13.5]	7.6% [3.3–14.2]	
5 years		14.3% [8.2–22.1]	9.1% [4.2–16.5]	
10 years		20.5% [12–30.6]	18.7% [8.7–31.8]	

a *Annualized event-rates (event per patient year = ppy)*.

b *Cumulative incidence with death as competing event*.

c *Chi-squared test*.

d *Fisher's exact test*.

e *Gray's test*.

### Mortality and Morbidity in Patients Aged 50–70 Years

**Figure 3:**
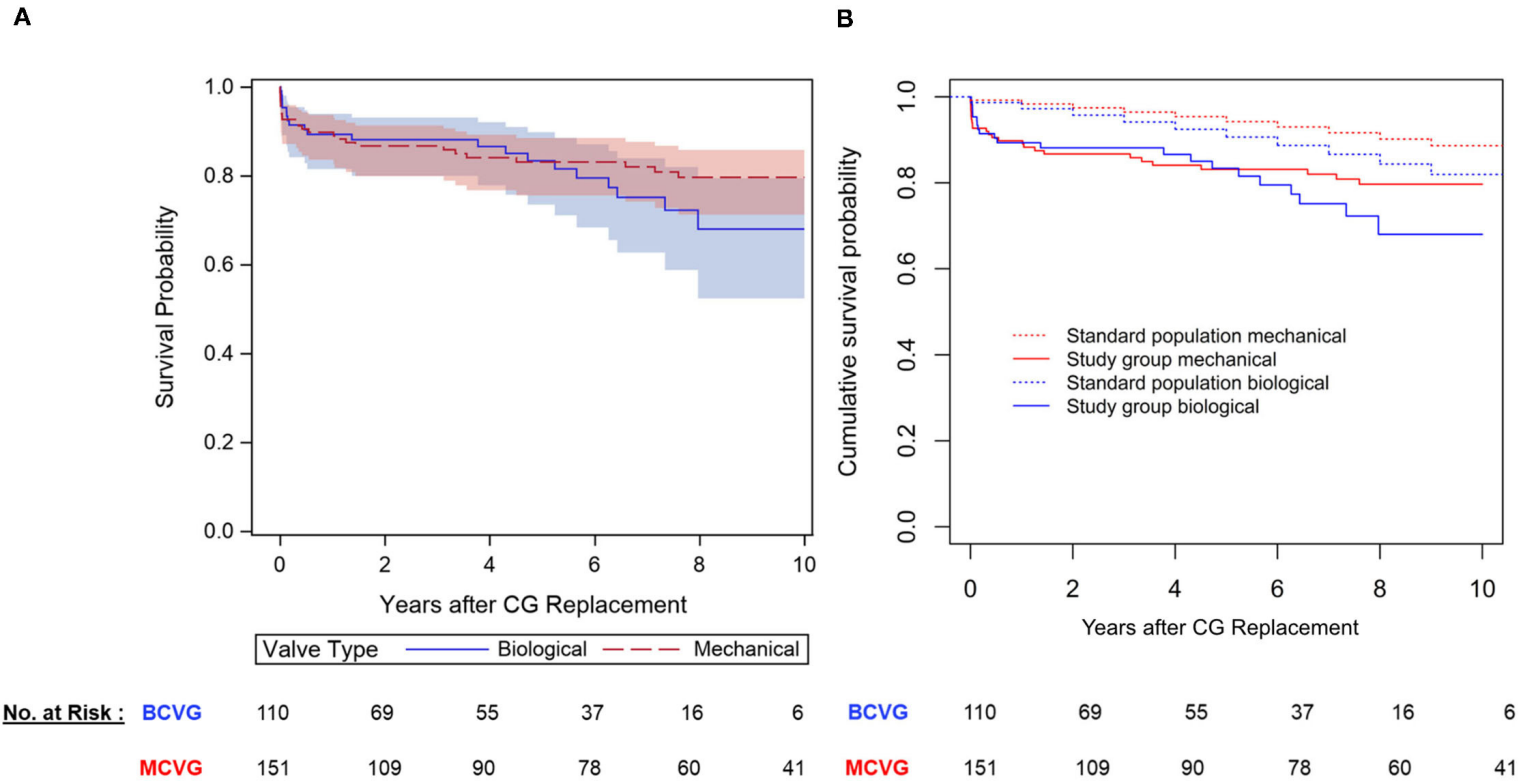
Survival in patients aged 50–70 years. **(A)** Kaplan-Meier (KM)-estimated survival stratified after valve type **(B)** KM-estimated survival of each valve type compared to a group-specific age- and sex-matched Austrian standard population.

In patients aged 50–70 years (*n* = 261; MCVG = 161, BCVG = 110), the 30-day mortality was 6.5% (*n* = 16) with no significant differences between valve types (MVCG *n* = 11, 6.8%, BCVG 4.5% *n* = 5, *p* = 0.36). The Kaplan-Meier estimated freedom from mortality at 1, 5, and 10 years was 89.8 ± 2.5, 83.1 ± 3.3, and 79.7 ± 3.7% in the mechanical cohort and 89.3 ± 3.1, 83.4 ± 4.1, and 68 ± 6.9% in the biological cohort, respectively, with no statistically significant differences (log-rank *p* = 0.419; Figure 3A). When compared to an age- and sex-matched Austrian standard population, freedom from mortality was significantly lower in the MCVG cohort at 2 (98.4 vs. 86.7%, *p* = 0.0004) and 5 years (95.4 vs. 83.1%, *p* = 0.0017), but not at 10 years (88.6 vs. 79.6%, *p* = 0.052). In the BCVG cohort, survival was significantly lower at 2 years (97.2 vs. 88.1%, *p* = 0.013), but not at 5 (92.4 vs. 83.4%, *p* = 0.06) or 10 years (81.9 vs. 68%, *p* = 0.17; Figure 3B).

**Figure 4:**
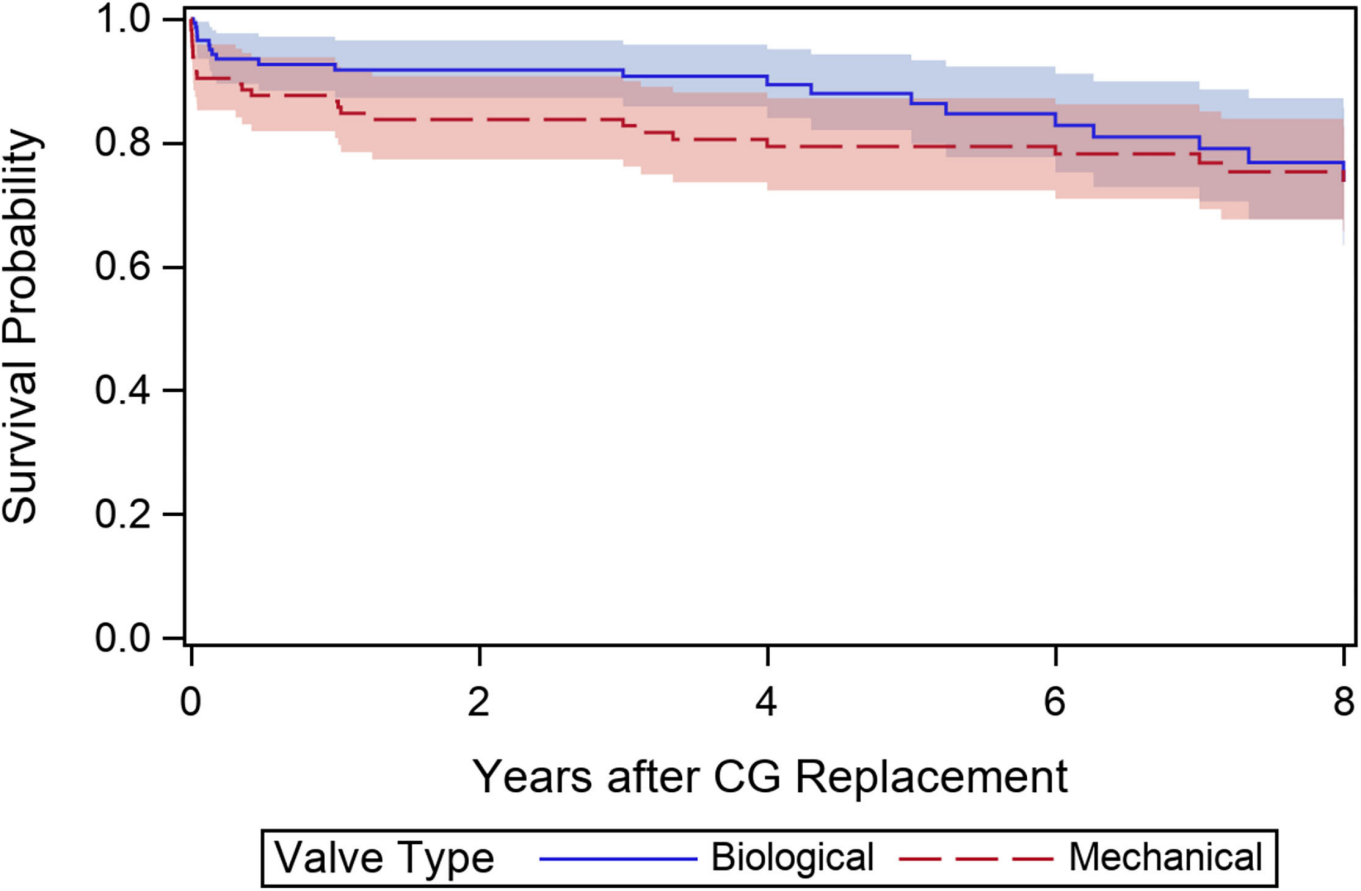
Direct adjusted survivor function (adjusted for age, EuroSCORE II, previous cardiac surgery, indication).

To investigate the effect of valve type on mortality in this subset, univariate and multivariable Cox proportional-hazards regression models were created. According to the number of observed events (*n* = 47), the four relevant prognostic confounders of age, EuroSCORE II (log_2_-transformed), previous cardiac surgery, and high-risk indication (aortic-dissection/endocarditis) were included in the multivariable model to evaluate the adjusted effect of valve type on survival. All variables except valve type were statistically significant prognostic factors for mortality following univariate analysis (Table 4). When adjusting for the confounders (multivariable model) and including the statistically significant time-varying effect of valve type (*p* = 0.034), the initially negative effect of the MCVG was found to later change to a decreased risk for mortality compared to the BCVG. However, the overall valve type effect remained statistically non-significant (*p* = 0.069). To illustrate the time-varying valve type effect, landmark analyses were performed at the landmark time points 2 and 5 years after CG replacement (Supplementary Material). A direct adjusted survivor function was created to illustrate this trend in patients aged 50–70 years (Figure 4). As a sensitivity analysis, a sub-analysis was performed on a 1:1 matched cohort based on patients age (categorized in 5-years increments), EuroSCORE II (categorized in deciles), and BAV (bicuspid vs. non-bicuspid valve) (Supplementary Material).

**Table 4:** Univariable and multivariable Cox regression models for post-operative mortality (50–70a).

**Prognostic factor**	**Univariable models**		**Multivariable model**	
	**HR [95% CI]**	***P*-value**	**HR [95% CI]**	***P*-value**
Age (years)	1.063 [1.01–1.12]	0.0127	1.07 [1.01–1.13]	0.02
EuroSCORE II (log_2_-transformed)	1.79 [1.42–2.17]	<0.0001	1.66 [1.29–2.14]	<0.001
Previous cardiac surgery	2.8 [1.39–5.65]	0.004	1.32 [0.611–2.86]	0.48
Indication (dissection/endocarditis)	2.98 [1.67–5.32]	0.0002	1.54 [0.79–3]	0.21
Valve type (mechanical)		0.079^b^		0.069^c^
6 months^a^	0.71 [0.39–1.29]		1.29 [0.64–2.58]	
1 year^a^	0.60 [0.32–1.13]		1.08 [0.52–2.24]	
5 years^a^	0.40 [0.17–0.94]		0.71 [0.28–1.80]	
9 years^a^	0.35 [0.14–0.90]		0.61 [0.22–1.70]	

a *After surgery*.

b *Overall p-value (time-dependent effect: p = 0.04)*.

c *Overall p-value (time-dependent effect: p = 0.033)*.

**Figure 5:**
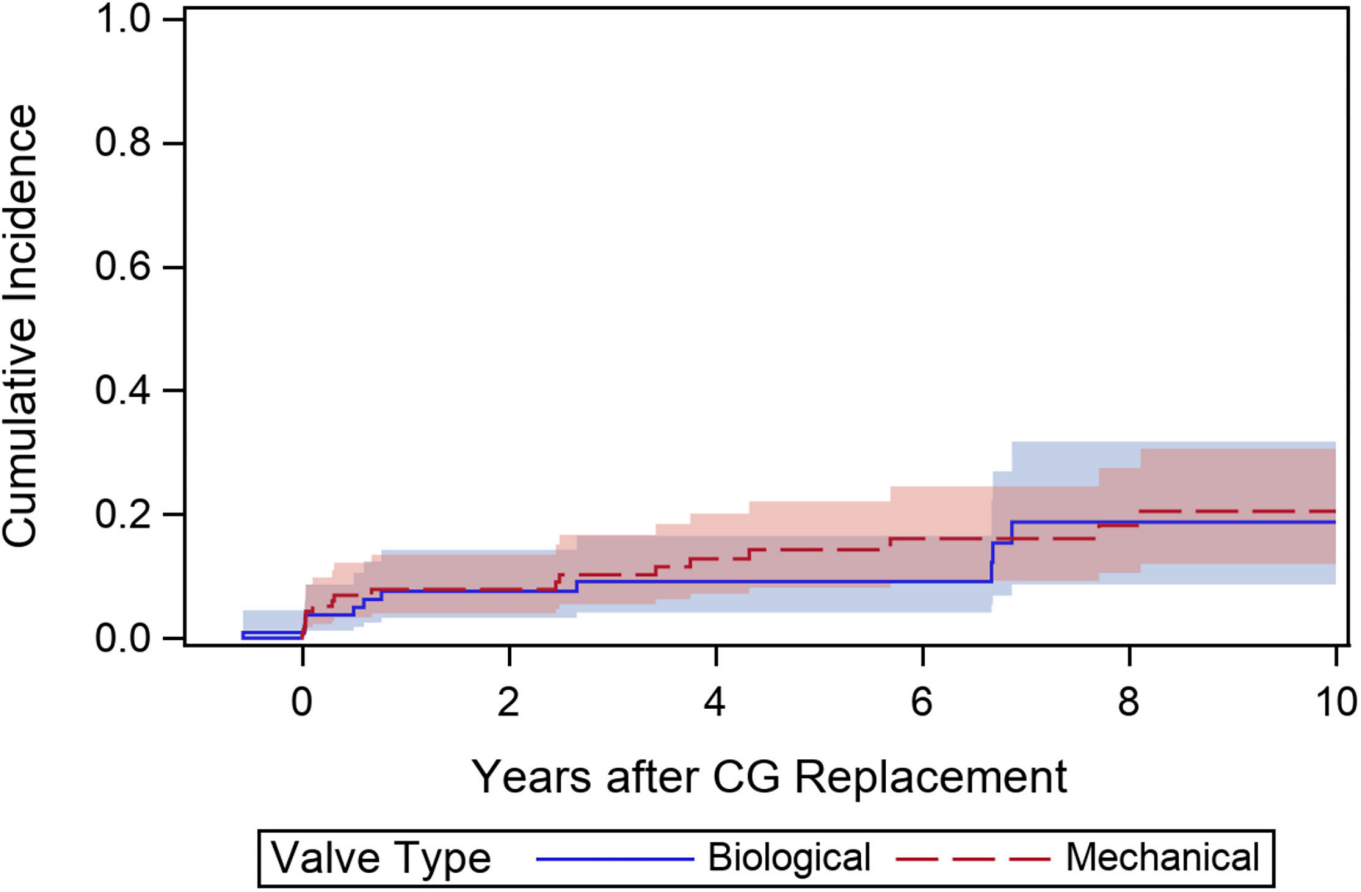
Cumulative incidence function for a composite endpoint for valve-related morbidity including bleeding events, embolic events, valve thrombosis, and structural valve deterioration in patients aged 50–70a.

To assess valve-related AEs, a composite endpoint incorporating bleeding, thrombosis, embolization, and SVD was assessed. A cumulative incidence function with death as competing event showed no differences between the two groups (*p* = 0.695) with a CI at 1, 5, and 10 years of 7.9 ± 2.4, 14.3 ± 3.6, and 20.5 ± 4.8% in the MCVG cohort and 7.6 ± 2.8, 9.1 ± 3.2, and 18.7 ± 6% in the BCVG cohort, respectively (Figure 5).

## Discussion

Within this study, we investigated long-term mortality and morbidity in a large single-center cohort of 507 patients who underwent CVG implantation after the modified Bentall procedure, and performed a specific subset analysis of patients between the ages of 50–70 years at index surgery.

Our study presents the following principal findings: (1) the modified Bentall procedure continues to represent the gold-standard for an all-comer cohort with aortic root pathologies and can be performed with excellent early mortality comparable to isolated aortic valve replacement (1.5%) in elective cases; (2) In the specific subset of patients aged 50–70 years, no clear survival benefit was shown for any type of prosthetic valve, however, there was a strong tendency for an initially higher risk with MCVG which decreased compared to BCVG at long term follow-up; (3) In patients aged 50–70 years, no difference in the incidence of a composite endpoint comprising valve-related morbidity was observed between mechanical and biological CVGs.

The observed 30-day mortality in our overall cohort was 5.9%, with no significant differences between mechanical and biological CVGs, which is comparable to other collectives in the literature. Di Marco et al. reported on the currently largest single-center cohort (*n* = 1,045) after MCVG and BCVG implantation, showing operative mortality of 5.3% without significant differences between valve types (3). The Leipzig group reported hospital mortality of 3.9% in their overall cohort of 597 patients who were implanted with MCVGs and BCVGs (4). In a different single-center cohort of 593 patients with only MCVGs, the 30-day mortality was 3.2%, with 2.5% in elective cases and 6.5% in urgent cases (5). A meta-analysis published in 2016, including 46 studies with 7,629 patients undergoing a Bentall procedure, showed a pooled early mortality of 6% (12). We observed low 30-day mortality of 1.5% in patients who underwent elective CVG implantation, which is comparable to isolated SAVR and represents an excellent safety profile for an aortic root procedure. The low mortality of elective cases might be attributable to the standardization of the procedure within the last 20 years including modifications, such as the button technique and the broad implementation of temporary mechanical circulatory support. However, in urgent/emergent cases and type-A dissection, the Bentall procedure remains at higher risk as indicated by the 30-day mortality in our collective and within the literature (3, 4, 13).

The optimal valve choice in the so-called “gray area” of patients aged 50–70 years has been a matter of discussion in past decades and remains controversial. Nevertheless, the trend toward an increasing number of bioprostheses used in SAVR continues and is also present in CVG replacement. In our series, 86.3% of patients between 2000 and 2010 received an MCVG, while BCVG implantation was more frequent between 2010 and 2020 with 53% (*p* < 0.001). Evidence on valve choice in the Bentall procedure is limited as only a few studies have investigated the effect of valve type, especially in patients aged 50–70 years. An earlier analysis, including BCVG, stent-less prostheses, and MCVG, showed no differences regarding early and midterm mortality between mechanical and biological solutions (6). In a more recent publication with two equally sized (*n* = 138) propensity-matched CVG cohorts stratified after valve type, no differences in early and late mortality were observed between the groups (7). Follow-up was limited with mean values ranging from 29 months in the biological group to 40 months in the mechanical group. In the only subgroup analysis of patients aged 50–70 years, Etz et al. reported no significant differences in long-term survival, reinterventions rates, or stroke (4). They concluded that BCVG might be an equivalent alternative to MCVG in this patient subset. However, their conclusion is challenged by more recent evidence from larger retrospective analyses in SAVR patients. Particularly, a California state-wide analysis showed improved survival in patients with mechanical AVR below the age of 55 and data from the nationwide SWEDEHEART registry reported a survival benefit of mechanical over biological prostheses in patients aged up to 69 years at replacement (14, 15). Even though we did not find a statistically significant effect of valve type on mortality, a time-varying effect with initially increased risk for MCVG which later decreased compared to BCVG was notable (*p* = 0.069). Similar results were observed in an Italian multicentric-registry comparing MCVG and BCVG implantation, where a non-significant (*p* = 0.09) trend to improved late survival in MCVG patients was reported (8). A possible factor contributing to the initially increased mortality in the MCVG cohort might be a numerically higher number of urgent (MCVG 11.9% vs. BCVG 10%) and emergent (MCVG 23.8% vs. BCVG 16.4%) procedures (nonetheless, surgical indication, including dissection and endocarditis, was not a predictive factor for mortality in the multivariable model). Patients in the BCVG group showed a trend toward decreased survival in long-term follow-up, which can be partially explained by an older cohort with a numerically higher burden of comorbidities (although statistically non-significant). Further considerations, such as the impact of structural deterioration remain speculative, as only one case of SVD and one root rereplacement in the BCVG aged 50–70a were observed in our cohort with no significant differences in reinterventions. Our cohort presented a median survival follow-up of 102.8 months in the MCVG group (57.8 months for AEs) and 55.1 months in the BCVG group (37.2 months for AEs). SVD, which remains the burden of bioprosthetic valves, usually occurs after 5 years postoperatively. As we recently showed, a rapid increase in SVD events in certain valve types can be expected starting 6 years after surgery with aortic valve reinterventions as a negative predictor of survival in SAVR (16). Longer follow-up times in this subset (50–70a) will be needed to obtain a more comprehensive picture of the effects of SVD in CVG replacement. The survival benefit of mechanical valves in patients who have undergone SAVR aged up to 55/69 years shown by recent analyses (14, 15) and the absence of evidence of survival benefits for BCVG should not be neglected when opting for the optimal CVG choice in patients aged 50–70 years.

Interestingly, the incidence of a composite endpoint for valve-related morbidity did not differ between the MCVG and BCVG cohorts aged 50–70 years (Figure 5). No significant difference in the number of bleeding or embolic events was observed between the groups. This might be attributable to improved management of anticoagulation in patients receiving mechanical prostheses, including self-measurement strategies and lower thrombogenicity of modern mechanical valves.

### Study Strengths and Limitations

This study presents one of the largest single-center cohorts of patients who underwent a modified Bentall procedure at a tertiary care center with a relevant number of patients aged 50–70 years at the time of surgery. Due to its retrospective nature, this study presents has some limitations. Although patient follow-up was meticulously performed, it was naturally hindered by patient refusal, relocation, and death. In a single-center cohort, a specific set of surgeons is represented and may thus introduce an unknown bias. Biological CVGs have been implanted more frequently in the latter half of the study period. Therefore, follow-up in this group is inevitably proportionally shorter compared to MCVGs. Consequently, analyses between the valve groups in patients aged 50–70 years were limited to 10 years of follow-up. The present study is retrospective in nature and, therefore, its results cannot substitute those of a prospective randomized trial which would randomize eligible patients to either mechanical or biological CVG replacement.

## Conclusion

The modified Bentall technique presents satisfactory results in an all-comer series with low mortality when electively performed, but remains a high-risk procedure when performed in urgent or emergent cases. The choice of valve conduit showed no statistically significant effects on mortality in patients aged 50–70 years. However, there was a strong tendency for an initially higher risk with MCVG, which decreased compared to BCVG at long-term follow-up. Further studies with longer follow-up of biological valve conduits are needed to determine the ideal choice of valve in this specific patient subset.

## Data Availability Statement

The raw data supporting the conclusions of this article will be made available by the authors, without undue reservation.

## Ethics Statement

The studies involving human participants were reviewed and approved by Ethical Committee of the Medical University of Vienna (ethical-board number 2311/2020; date of approval 19.01.2021). The patients/participants provided their written informed consent to participate in this study.

## Author Contributions

PW was responsible for conceptualization, data curation, investigation, formal analysis, methodology, and writing. JG and IC were responsible for data curation, resources, review, and editing. AKa was responsible for the methodology, resources, and formal analysis. EO, SM, and M-ES were responsible for reviewing, editing, and conceptualization. AKo and GL were responsible for the review with editing and supervision. MA was responsible for conceptualization, review with editing, and supervision. ME was responsible for conceptualization, project administration, reviewing, editing, and supervision. All authors contributed to the article and approved the submitted version.

## Conflict of Interest

MA is a proctor, speaker, consultant in Abbott, Edwards, Medtronic, and received institutional funding (Abbott, Edwards, Medtronic, LSI). AKo is a proctor (Edwards) and received speaker fees (Edwards). GL is an advisory-board member (Edwards). The remaining authors declare that the research was conducted in the absence of any commercial or financial relationships that could be construed as a potential conflict of interest.

## Publisher's Note

All claims expressed in this article are solely those of the authors and do not necessarily represent those of their affiliated organizations, or those of the publisher, the editors and the reviewers. Any product that may be evaluated in this article, or claim that may be made by its manufacturer, is not guaranteed or endorsed by the publisher.

## References

[1] Bentall H De Bono A. A technique for complete replacement of the ascending aorta. Thorax. (1968) 23:338–9. 10.1136/thx.23.4.338 5664694 PMC471799

[2] Kouchoukos NT Wareing TH Murphy SF Perrillo JB. Sixteen-year experience with aortic root replacement. Results of 172 operations. Ann Surg. (1991) 214:308–18; discussion 18–20. 10.1097/00000658-199109000-00013 1834031 PMC1358653

[3] Di Marco L Pacini D Pantaleo A Leone A Barberio G Marinelli G. Composite valve graft implantation for the treatment of aortic valve and root disease: results in 1045 patients. J Thorac Cardiovasc Surg. (2016) 152:1041–8.e1. 10.1016/j.jtcvs.2016.05.021 27312787

[4] Etz CD Bischoff MS Bodian C Roder F Brenner R Griepp RB. The Bentall procedure: is it the gold standard? A series of 597 consecutive cases. J Thorac Cardiovasc Surg. (2010) 140 (6 Suppl):S64–70; discussion S86–91. 10.1016/j.jtcvs.2010.07.033 21092800

[5] van Putte BP Ozturk S Siddiqi S Schepens MA Heijmen RH Morshuis WJ. Early and late outcome after aortic root replacement with a mechanical valve prosthesis in a series of 528 patients. Ann Thorac Surg. (2012) 93:503–9. 10.1016/j.athoracsur.2011.07.089 22200369

[6] Byrne JG Gudbjartsson T Karavas AN Mihaljevic T Phillips BJ Aranki SF. Biological vs. mechanical aortic root replacement. Eur J Cardiothorac Surg. (2003) 23:305–10. 10.1016/s1010-7940(02)00816-3 12614798

[7] Pantaleo A Murana G Di Marco L Jafrancesco G Barberio G Berretta P. Biological versus mechanical Bentall procedure for aortic root replacement: a propensity score analysis of a consecutive series of 1112 patients. Eur J Cardiothorac Surg. (2017) 52:143–9. 10.1093/ejcts/ezx070 28407120

[8] Lechiancole A Celiento M Isola M Gatti G Melina G Vendramin I. Modified Bentall procedure: mechanical vs biological valved conduits in patients older than 65years. Int J Cardiol. (2019) 296:38–42. 10.1016/j.ijcard.2019.07.053 31351789

[9] Akins CW Miller DC Turina MI Kouchoukos NT Blackstone EH Grunkemeier GL. Guidelines for reporting mortality and morbidity after cardiac valve interventions. Ann Thorac Surg. (2008) 85:1490–5. 10.1016/j.athoracsur.2007.12.082 18355567

[10] Schemper M Smith TL. A note on quantifying follow-up in studies of failure time. Control Clin Trials. (1996) 17:343–6. 10.1016/0197-2456(96)00075-X 8889347

[11] *Statistics, Austria*. Available online at: https://www.statistik.at/web_de/statistiken/menschen_und_gesellschaft/bevoelkerung/sterbetafeln/index.html

[12] Mookhoek A Korteland NM Arabkhani B Di Centa I Lansac E Bekkers JA. Bentall procedure: a systematic review and meta-analysis. Ann Thorac Surg. (2016) 101:1684–9. 10.1016/j.athoracsur.2015.10.090 26857635

[13] Yang B Patel HJ Sorek C Hornsby WE Wu X Ward S. Sixteen-Year experience of david and bentall procedures in acute type a aortic dissection. Ann Thorac Surg. (2018) 105:779–84. 10.1016/j.athoracsur.2017.09.029 29258677

[14] Goldstone AB Chiu P Baiocchi M Lingala B Patrick WL Fischbein MP. Mechanical or biologic prostheses for aortic-valve and mitral-valve replacement. N Engl J Med. (2017) 377:1847–57. 10.1056/NEJMoa1613792 29117490 PMC9856242

[15] Glaser N Jackson V Holzmann MJ Franco-Cereceda A Sartipy U. Aortic valve replacement with mechanical vs. biological prostheses in patients aged 50-69 years. Eur Heart J. (2016) 37:2658–67. 10.1093/eurheartj/ehv580 26559386

[16] Werner P Coti I Kaider A Gritsch J Mach M Kocher A. Long-term durability after surgical aortic valve replacement with the trifecta and the intuity valve—a comparative analysis. Euro J Cardio Thorac Surg. (2021) 61:416–24. 10.1093/ejcts/ezab470 34738111

