# Supplementary material for: Long Term Results of the Modified Bentall Procedure With Mechanical and Biological Composite Valve Grafts
Source: Front Cardiovasc Med. 2022 Apr 6;9:867732. doi: 10.3389/fcvm.2022.867732 (PMC9019133; doi:10.3389/fcvm.2022.867732)
Supplement: Supplementary file 1 [file Data_Sheet_1.PDF]

Supplemental material

As a sensitivity analysis, a sub-analysis (of patients aged 50-70a) was performed on a 1:1 matched cohort based on patients age (categorized in 5-years increments), EuroSCORE II (categorized in deciles) and BAV (bicuspid vs non-bicuspid valve). The survival probabilities of the 45 matched valve type pairs were estimated by the Kaplan-Meier method and compared using the log-rank test (accounting for the paired situation by stratification).

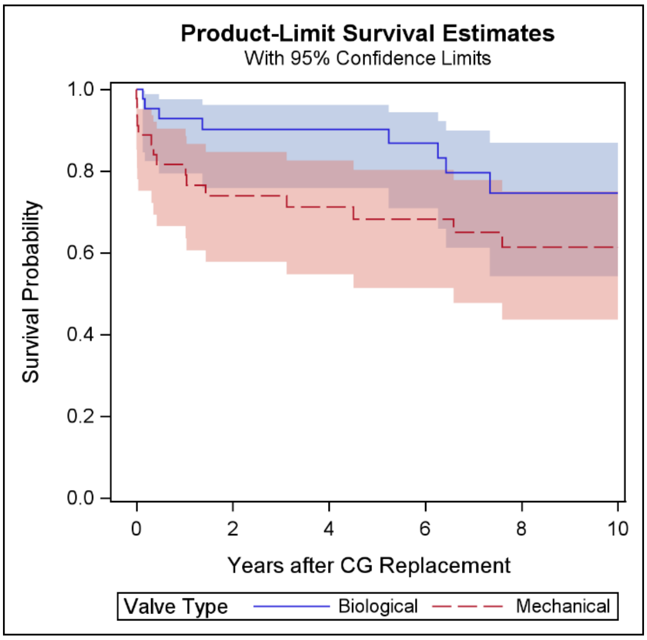

<u>Supplemental Figure 1:</u> Survival of an age-, EuroSCORE II- and BAV-matched cohort in the subgroup of patients aged 50-70a (n=45 matched pairs); p=0.046

To illustrate the time-varying valve type effect, landmark analyses were performed at the landmark time points two and five years after CG replacement in patients aged 50-70 years. Thereby, only patients still at risk at two and five years, respectively, were included in the analyses. The respective landmark time points were set to time 0, and survival after this time points were described by Kaplan-Meier curves.

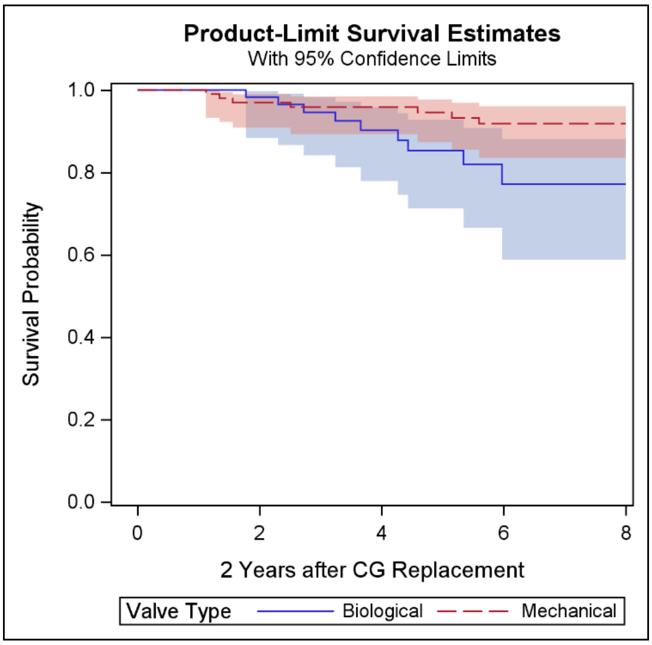

<u>Supplemental Figure 2:</u> Landmark analysis: Survival probability 2 years after CG replacement of patients aged 50-70 years (MCVG: n=109; BCVG: n=69)

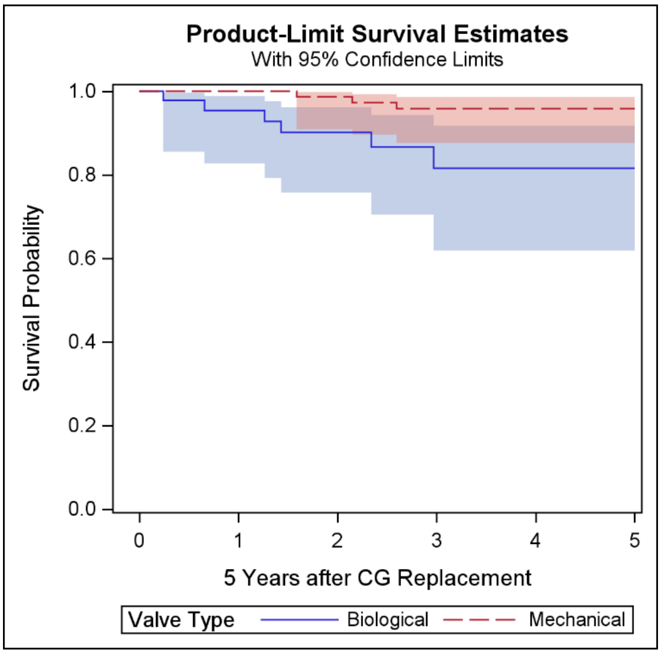

<u>Supplemental Figure 3:</u> Landmark analysis: Survival probability 5 years after CG replacement of patients aged 50-70 years (MCVG: n=84; BCVG: n=49)
